# The Effects of Multiple Micronutrient Fortified Beverage and Responsive Caregiving Interventions on Early Childhood Development, Hemoglobin, and Ferritin among Infants in Rural Guatemala

**DOI:** 10.3390/nu15092062

**Published:** 2023-04-25

**Authors:** Alysse J. Kowalski, Victor Alfonso Mayen, Silvia de Ponce, Kaley B. Lambden, Nick Tilton, Lisa M. Villanueva, Ana M. Palacios, Gregory A. Reinhart, Kristen M. Hurley, Maureen M. Black

**Affiliations:** 1 Department of Pediatrics, University of Maryland School of Medicine, Baltimore, MD 21201, USA; akowalski@som.umaryland.edu; 2 Asociación para la Prevención y Estudio del VIH/Sida, Retalhuleu 11001, Guatemala; valfonso@apevihs.org (V.A.M.); shdepster@gmail.com (S.d.P.); 3 Department of International Health, Johns Hopkins Bloomberg School of Public Health, Baltimore, MD 21205, USA; kaley.lambden@gmail.com (K.B.L.);; 4 Department of Epidemiology and Public Health, University of Maryland School of Medicine, Baltimore, MD 21202, USA; natilton@gmail.com; 5 The Mathile Institute for the Advancement of Human Nutrition, Dayton, OH 45414, USA; lisa@chispuditos.com (L.M.V.); apalacios@georgiasouthern.edu (A.M.P.); reinhart.greg@gmail.com (G.A.R.); 6 Jiann Ping Hsu College of Public Health, Georgia Southern University, Statesboro, GA 30458, USA; 7 RTI International, Research Triangle Park, NC 27709, USA

**Keywords:** multiple micronutrient supplementation, responsive caregiving, socioemotional development, early child development, ferritin, hemoglobin, infant, Guatemala

## Abstract

Undernutrition and a lack of learning opportunities can jeopardize long-term growth and development among infants in low- and middle-income countries. We conducted a 6-month 2 × 2 cluster-randomized trial to assess the effects of multiple micronutrient-fortified beverages and responsive caregiving interventions among infants 6–18 months in 72 community sectors in southwest Guatemala. We administered baseline and endline assessments of childhood development (Bayley Scales of Infant and Toddler Development) and socioemotional development (Brief Infant Toddler Socio-Emotional Assessment) and measured ferritin and hemoglobin on a subsample. The trial was analyzed using linear mixed models. At the baseline, the mean age (SD) was 13.0 (4.6) months, including 49% males, 32% who were stunted, 55% who were anemic, and 58% who were iron deficient. At the endline (*n* = 328/386, 85% retention), there was no synergistic effect on the fortified beverage and responsive caregiving intervention. Compared to the non-fortified beverage group, socioemotional development improved in the fortified beverage group. There were no intervention effects on other measures of child development, hemoglobin, or ferritin. In a setting with high rates of anemia and iron deficiency, a multiple micronutrient-fortified beverage improved infants’ socioemotional development.

## 1. Introduction

Early childhood development lays the foundation for subsequent learning throughout the life course and is associated with health, schooling attainment, and earning potential, and with individual, family, and societal implications [1,2]. Estimates of over 250 million young children in low- and middle-income countries (LMICs) are not reaching their developmental potential, largely due to undernutrition and a lack of early learning opportunities, jeopardizing their long-term growth and development [3,4]. To support early development, children need nurturing care, including health, nutrition, safety and security, and opportunities for early learning in the context of responsive caregivers and a stable home [1,5].

Healthy brain development includes dendritic branching, synapse maturation and function, and myelination is dependent on adequate nutrition, including multiple micronutrients [6]. Globally, over half of young children are deficient in at least one micronutrient, with the prevalence of maternal and child micronutrient deficiencies ranging from an estimated 9% for zinc deficiency to 20% for iron deficiency [7,8]. Dietary intake in LMICs is often inadequate to meet micronutrient requirements. Multiple micronutrient supplementation is effective in improving serum micronutrient concentrations and in reducing rates of anemia [9,10], but its effects on children’s development are understudied.

Responsive caregiving refers to caregivers’ prompt, age-appropriate, and sensitive response to children’s behavior [11,12]. Positive experiences for caregivers and children are mutually reinforcing and promote continued interactions, supporting attachment and self-regulation [13]. Applied to feeding, responsive caregiving is a culturally adaptable strategy that encourages children to eat autonomously and in response to physiological and developmental needs, thereby building self-regulation [14]. Applied to play, responsive caregiving interventions include early learning in a responsive and nurturant context which build parents’ knowledge, attitudes, and practices related to responsive care, with positive effects on children’s cognitive, language, psychosocial, and motor development [15,16]. 

Integrating nutrition and responsive caregiving interventions may be an efficient use of resources to promote early childhood development. Meta-analyses and systematic reviews of combined interventions have shown specificity, with beneficial effects on nutrition attributable to nutrition components and beneficial effects on development attributable to psychosocial components, with few investigations of synergistic effects [17,18,19]. 

Guatemala provides a unique opportunity to investigate the impact of combined nutrition and responsive caregiving interventions. Guatemala has one of the highest rates of undernutrition seen globally, and the highest in Latin America. Over 45% of children < 5 years are stunted (height-for-age *z*-scores < −2), with higher rates among rural and indigenous communities, reflecting inequity and poverty [20]. Stunting, an indicator of linear growth failure, is a marker of general environmental deprivation characterized by inadequate sanitation, food insecurity, and other aspects of poverty [21]. In such environments, micronutrient deficiencies are common and opportunities for early learning may be limited. 

The objective of this study was to evaluate the independent and synergistic effects of multiple micronutrient supplementation and responsive caregiving interventions on young children’s development and nutritional status in rural Guatemala. The study was designed to test three hypotheses: (1) multiple micronutrient-fortified beverage groups will have better development and nutritional status compared to placebo groups, (2) responsive caregiving groups will have better development than groups without responsive caregiving, and (3) the combined multiple micronutrient-fortified beverage and responsive caregiving group will have better development than the other three groups, attributed to synergistic effects. 

## 2. Materials and Methods

In collaboration with the non-governmental organization APEVIHS (Association for the Prevention and Study of HIV and AIDS, Spanish acronym) located in western Guatemala, we conducted a study, titled Mejorando la Inteligencia en la Niñez del Takalik Abaj (Improving Childhood Intelligence of the Takalik Abaj), a reference to a Mayan archeological site of cultural significance to the communities. We evaluated two home-based interventions: multiple micronutrient-fortified beverages and responsive caregiving coaching, singly and in combination, with regard to development and nutrition biomarkers of infants 6–18 months (the focus of this study) and preschoolers 36–52 months (the focus of a separate study). 

### 2.1. Site and Study Design

We conducted a cluster-randomized controlled trial in the Department of Retalhuleu from 2015 to 2017. The department is located in southwestern Guatemala and extends from the mountains to the Pacific Ocean. The area was selected due to the high prevalence of stunting (39% in children 6–72 months), suggesting nutritional inadequacies and food insecurity [22]. A rural municipality with proximity to an urban center was selected as the study site. In 2018, the municipality had a population of 41,000 [23]. The municipality was organized into smaller units of communities and locally recognized sectors. We approached and shared information with 32 communities (representing 77 sectors), and all expressed interest in participating in the study. Four communities were subsequently excluded due to community violence or agriculturally driven seasonal fluctuations in population size. 

A 2 × 2 factorial design was used to efficiently test the multiple micronutrient-fortified beverage and responsive caregiving interventions and their interaction [24]. Treatments were allocated at the sector level to reduce contamination and a stratification procedure was used to reduce the risk of imbalance. Using Guatemalan census data, we characterized sectors with respect to size, distance from a highway, and access to a health center. We organized non-contiguous sectors sharing common characteristics into fourteen strata of at least four sectors per group. Sectors were allocated to treatments in two randomization steps within each stratum: multiple micronutrient supplementation or placebo and responsive caregiving or no caregiving intervention, yielding four treatment groups: (1) fortified beverage only, (2) responsive caregiving + placebo, (3) fortified beverage + responsive caregiving, and (4) placebo + no responsive caregiving (control). The researchers and evaluation team were unaware of treatment assignments and supplement classification (fortified beverage/placebo). Home visitors and participants were unaware of beverage classification. 

### 2.2. Sample Size

We a priori determined the sample size needed to evaluate the primary outcome, childhood development. Presuming 32 communities with an average size of 15 infants and assuming a moderate effect size on childhood development (0.5 SD) using 2-sided tests (α = 0.05, 80% power) with 85% retention, an intraclass correlation of 0.03, and repeated measures correlation of 0.4, the study required a sample size of 480 infants.

### 2.3. Ethical Approval

We obtained ethical approval from the Universidad Francisco Marroquín and the Institution Review Board of the University of Maryland School of Medicine. The trial was registered at ClinicalTrials.gov (NCT02302729, registered on 27 November 2014). 

### 2.4. Recruitment

After obtaining approval from municipality leaders, surveillance was conducted in the targeted communities to identify potential participants. Home visitors recruited parents of infants 6–18 months (97% mothers, 3% maternal grandmothers). To focus on children at nutritional risk, we prioritized children with length-for-age *z*-score (LAZ) < −1. Inclusionary criteria were Spanish-speaking and an intention to remain in the area for the coming year. Exclusionary criteria were severe undernutrition (length- or weight-for-age *z* < −3) or chronic illness or disabling conditions that could interfere with growth and/or development. Children with severe undernutrition were referred for medical treatment. Parents provided informed consent for themselves and their infants.

### 2.5. Multiple Micronutrient-Fortified Beverage/Placebo Interventions

Atoles are traditional beverages, typically prepared from masa corn flour, that are widely consumed throughout Central America. Chispuditos^®^ was a corn-soy blend fortified with 21 micronutrients (i.e., iron, zinc, and B vitamins) that was consumed as a beverage (see Table 1). Chispuditos^®^ was developed by a team of nutrition scientists at the Mathile Institute for the Advancement of Human Nutrition and manufactured locally. Pre-post-acceptability studies with young children suggest high acceptance and daily adherence [22,25,26,27].

The placebo for the study consisted of the same corn-soy blend fortified only with vitamin B2 (which has no known effect on the outcomes) that was otherwise identical to Chispuditos^®^. The two products were supplied in identical packets with manufacturer-assigned codes to differentiate the multiple micronutrient supplements from the placebo. Codes were stored in a sealed envelope at the Mathile Institute. Products were not commercially available and were provided to participating families free of charge. 

Home visitors were scheduled to visit households at 3-week intervals for 6 months, where they delivered 1 lb bags of Chispuditos^®^/placebo (24 servings) in quantities sufficient for other children in the household and provided nutrition counseling. Mothers were instructed to serve Chispuditos^®^/placebo to the target infant daily in addition to typical meals. 

### 2.6. Responsive Caregiving Intervention

The responsive caregiving intervention utilized parent coaching adapted from the WHO/UNICEF Care for Child Development package that uses play and communication to promote responsive caregiving activities [28]. Home visitors incorporated age-appropriate behavior messages into the coaching intervention using hand-held flipcharts with local examples on one side and notes for the home visitor on the other (see Table 2). The 6-session infant curriculum emphasized responsive caregiving applied to both feeding and play and learning. Responsive feeding included responding to children’s cues of hunger and satiety in a prompt, emotionally supportive, contingent, and developmentally appropriate manner, ensuring that the feeding context is pleasant, with verbal and non-verbal encouragement and modeling during feeding [11]. Responsive caregiving during play and learning included providing opportunities to play and explore while following the child’s lead through imitation and responding. Home visitors reinforced the key messages using questioning to check for understanding, demonstration, and practice activities with mothers and children, and motivational strategies such as goal-setting activities, praise and positive feedback, and problem-solving [29]. Coaching sessions lasted approximately 20 min, with each session reviewing the previous lesson’s material before introducing new concepts. 

### 2.7. Training and Intervention Delivery

Enrollment and intervention delivery were conducted by home visitors who lived in the study communities. Home visitors participated in a 7-day training led by the research team on nutrition counseling, parental coaching, informed consent, and data entry using a combination of didactic instruction and role-playing. Due to safety concerns, all home visitors were male and reached the communities via motorbike. A supervisor with extensive field experience and a background in nutrition supervised the intervention delivery. 

Home visitors were scheduled to visit households at 3-week intervals for 6 months, where they delivered the interventions. Due to election-related violence in the communities, the study visits were curtailed at times and extended over 8 months, with a doubling of visits when necessary. 

### 2.8. Evaluation

Evaluations were conducted at the baseline prior to randomization and the endline (six months post-baseline). The outcome measures included standardized measures of child development, socioemotional development, and nutrition biomarkers. Questionnaires addressed family demographics and social and environmental factors and were either validated in Spanish-speaking populations or adapted, translated, and/or developed for Guatemala. 

Participants were transported to a centralized site for evaluations, ensuring that evaluators were unaware of community-level assignments. The evaluation team received specialized training to conduct direct assessments of children’s development and anthropometry. Most evaluators had a background in teaching; all had experience working with children. The evaluation team was overseen by a licensed psychologist from Guatemala. Data were collected on tablets using the mobile data collection software Magpi^®^ by DataDyne LLC (Washington, DC, USA) and uploaded from the field office daily. 

### 2.9. Outcome Measures

#### 2.9.1. Child Development

Infant development was measured at the baseline and the endline using the Bayley Scales of Infant and Toddler Development-III Spanish, yielding scores on cognitive, language, and motor development [30]. Infant social and emotional behaviors were assessed using the BITSEA (Brief Infant Toddler Social-Emotional Assessment), yielding scores on socioemotional competence and problems [31]. 

#### 2.9.2. Nutrition Biomarkers 

A contracted phlebotomist collected blood from a subsample of infants at the baseline and the endline, which was chosen using a randomization procedure to assess changes in hemoglobin and ferritin concentrations. Ferritin concentration reflects iron stores and hemoglobin concentration measures the red blood cell supply and is affected by multiple determinants. Acute phase proteins C-reactive protein and alpha-1 acid glycoprotein were assayed and used to correct ferritin for the effects of inflammation [32]. Ferritin was right-skewed, and log transformed prior to analysis.

### 2.10. Control Measures 

Data on household, maternal, and child characteristics were assessed at the baseline to examine their potential influence on the outcome measures. Data were gathered on social factors and environmental conditions, including family ethnicity and household food security using the Household Food Insecurity Access Scale [33]. Socioeconomic status was assessed by household asset ownership. Data were gathered on maternal age, marital status, and schooling attainment. Child weight and length were measured using Seca scales and Schorr length boards following a standardized protocol. Measurements were collected in triplicate and averaged. Weight-for-age, length-for-age, weight-for-length, and BMI-for-age were converted to *z*-scores using WHO standards [34]. 

### 2.11. Statistical Analysis

For all outcomes, we conducted an intent-to-treat analysis using linear mixed-effects regression models with random intercepts for community and subject to account for the clustered design and baseline scores, respectively. To assess the synergistic effects of receiving both the fortified beverage and responsive caregiving interventions on measures of early childhood development, we included a 3-way interaction term between each intervention and time. A significant 3-way interaction would indicate a multiplicative effect between the two interventions and the interaction term would be retained. If the 3-way interaction was nonsignificant, the interaction term would be dropped, and the effects of each intervention would be reported independently. Analyses were conducted using R v4.2.0 [35], with significance at *p* < 0.05. 

## 3. Results

### 3.1. Baseline Characteristics

**Figure 1:**
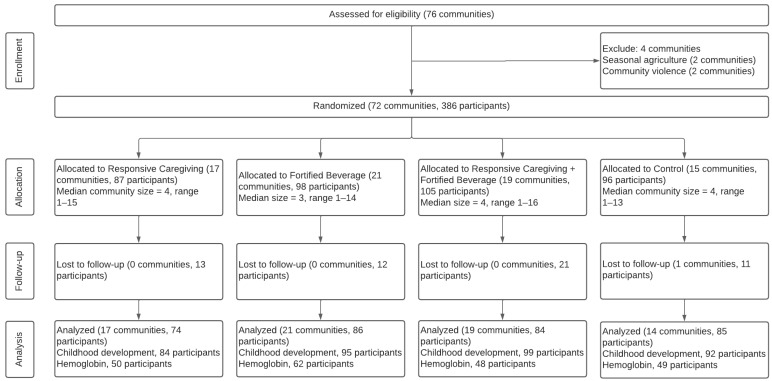
A cluster-randomized control trial CONSORT diagram.

The infant sample included 386 infants from 72 sectors (Figure 1). There were no significant baseline differences in household, maternal, child characteristics, or child development measures. The mean (SD) age of mothers was 27.5 (8.1), and 85% were married or in a relationship (Table 3). Most mothers (81%) had completed primary school or beyond. Over half (59%) of families were food-insecure at the baseline and 16% identified as an indigenous. Children were an average of 13.0 (4.6) months at enrollment, and 49% were boys. Stunting prevalence was 32%, 3% were wasted, and 19% were overweight or obese. At the endline, 329/386 children (85%) were retained. Retained children had significantly higher motor development scores and lower LAZ at the baseline. Children lost to follow-up were more likely to come from indigenous families. 

Blood specimens were collected from a subsample of 209 infants (54%). Compared to infants without a blood sample, infants with a blood sample had significantly higher scores (range: 3–4 points) on the child development assessments at the baseline. Maternal and household characteristics did not differ between infants with and without blood samples. Hemoglobin did not differ between treatment groups at the baseline. Median (IQR) ferritin was significantly lower among the responsive caregiving + fortified beverage group at the baseline. The prevalence of anemia and iron deficiency was 55% and 58%, respectively. 

### 3.2. Intervention Effects

The inclusion of a 3-way interaction term between the fortified beverage intervention, responsive caregiving intervention, and time did not significantly improve model fit, indicating that there was not a multiplicative effect of receiving both interventions on measures of early childhood development (see Table 4 footnote). The 3-way interaction term was, therefore, removed, and intervention effects were examined between intervention and no intervention groups.

Table 4 displays the observed mean (SD) baseline and endline values for measures of early child development and nutrition biomarkers in the fortified beverage, non-fortified beverage, responsive caregiving, and no responsive caregiving groups, as well as the difference in the change from the baseline to the endline for each outcome, comparing the fortified beverage to the non-fortified beverage group and the responsive caregiving to the no responsive caregiving group.

#### 3.2.1. Fortified Beverage Intervention Effects

**Figure 2:**
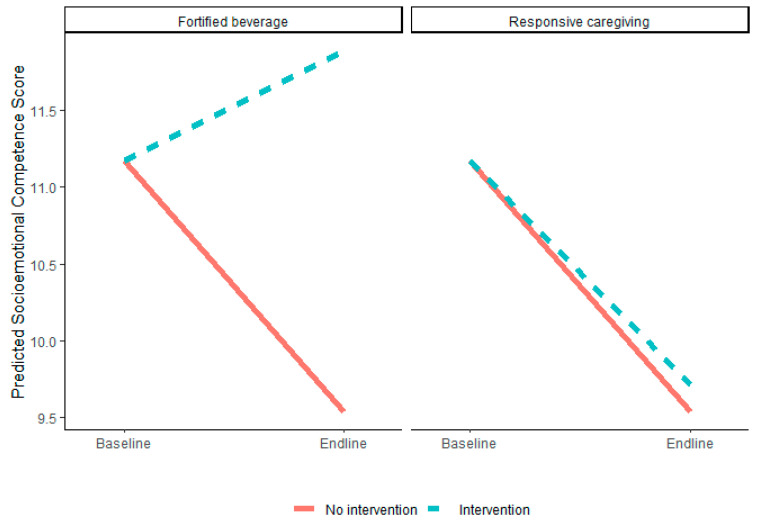
Predicted changes in socioemotional competence score from the baseline to the endline by intervention group.

Baseline to endline changes in socioemotional competence significantly differed between the fortified and non-fortified groups (*p* = <0.01), such that socioemotional competence increased in the fortified beverage group and declined in the non-fortified beverage group over time (fortified beverage vs. no fortified beverage = 2.34 [95% CI: 0.98, 3.7], Figure 2). Changes in cognitive, language, motor, and socioemotional problem scores and hemoglobin and ferritin did not differ between the fortified and non-fortified beverage groups.

#### 3.2.2. Responsive Caregiving Intervention Effects

Changes in the measures of early childhood development, hemoglobin, and ferritin from the baseline to the endline did not differ between the responsive caregiving and no responsive caregiving groups. 

## 4. Discussion

In a cluster-randomized controlled trial of fortified beverages and responsive caregiving interventions among infants at risk of micronutrient deficiencies in western Guatemala, there was no synergistic effect of the combined interventions on measures of child development or nutrition biomarkers. A receipt of the multiple micronutrient-fortified beverage improved infants’ socioemotional development. There was no effect of the responsive caregiving intervention.

Our finding that the fortified beverage was beneficial to socioemotional development is consistent with the findings from a systematic review and meta-analysis that showed that multiple micronutrient supplementation was positively associated with socioemotional scores in young children < 5 years [19]. More specifically, iron deficiency has been associated with non-adaptive socioemotional behavior including shyness and decreasing soothability, affect, and engagement [36]. These behaviors can interfere with the play and interactions that promote infant development.

We found no difference between the placebo and fortified beverage groups with respect to cognitive, language, and motor development, which was consistent with findings from other studies. In a trial of 3300 infants (age 8 + 0.3 months) in Bangladesh, infants received iron syrup, multiple micronutrient powders, or a placebo daily for 3 months [37]. Iron status and anemia improved, with no effects on the Bayley Scales of Infant Development scores following the intervention or at the 9-month follow-up and no impact on any other developmental or growth outcomes. In a systematic review of iron supplementation from 6 to 24 months, there was no clear relation between iron status and developmental outcomes up to age 4 [38]. 

Ferritin improved in each treatment group, while hemoglobin remained unchanged, which was inconsistent with a systematic review that found that ferritin and hemoglobin increased in response to multiple micronutrient supplementation [10]. The placebo group reflects the secular trend in ferritin during the study period. Similar increases in ferritin in the fortified beverage group might suggest that adherence to the fortified beverage/placebo intervention was suboptimal. While the fortified beverage/placebo was provided in quantities sufficient for each child in the family, atoles are common foods, and the study beverage may have been consumed by other children or family members. Adherence may also have been negatively impacted by disruptions to the home visits related to election violence, which has been widespread since the country’s transition to democracy in the mid-1980s [39]. Alternatively, both the fortified beverage and placebo groups received nutrition education counseling, which has been shown to improve minimum dietary diversity and minimum acceptable diet in young children in Guatemala [40]. 

Parenting programs that encourage nurturing care have been shown to have positive effects on children’s cognitive, language, and motor development in meta-analyses; effects on socioemotional development are inconclusive [15,16]. Effects were greater in LMICs and vulnerable groups, such as rural communities and caregivers with low education levels [15,16]. Programs that focused on responsive caregiving had greater effects on parenting knowledge, practices, and parent–child interactions with benefits to cognitive development [15]. Despite empirical evidence for responsive caregiving interventions, our study did not find an effect on children’s development. 

There are several possible explanations for this lack of findings. First, periodic violence required the study to be paused several times, which disrupted intervention fidelity and may have increased stress for families. To compensate for missed visits, home visitors were instructed to combine lessons which may have resulted in lessons being rushed or too many messages at one time for caregivers to absorb and implement [41]. A second contributing factor may have been that the home visitors were male, which given the widespread cultural context of machismo that focuses on masculine pride with little attention to caregiving [42], may have limited the effectiveness of the responsive caregiving intervention, which relies on rapport building and coaching with primary caregivers, which in Guatemala are primarily mothers. 

Given the lack of effect of responsive caregiving intervention, it is perhaps unsurprising that we did not observe a synergistic effect of combining the fortified beverage and responsive caregiving on children’s development, as hypothesized. Although combined interventions have positively affected development, the effect sizes tend not to differ from caregiving interventions alone [17,18]. While the theoretical basis for combining nutrition and caregiving interventions is strong and pragmatic from a program delivery standpoint, as both target young children during periods of rapid growth and development, bundling interventions may diminish the quality of the individual components by increasing the workload of the home visitor or overloading the recipient with information. Additional research is needed to ensure that caregiving and nutrition interventions are integrated such that home visitors can introduce them in a seamless manner that is respectful of caregivers’ time and cultural context.

Strengths of the study include high participant retention (85%). The intervention was delivered through a successful partnership with a local non-governmental organization and used a locally produced, culturally accepted beverage to deliver micronutrients. The study measured multiple aspects of children’s development using direct observation and maternal reports and objective measures of nutritional status. Limitations include disruptions to the fieldwork, which prevented the intervention from being implemented as designed. The lack of process monitoring data limits our ability to understand the uptake of the fortified beverage/placebo and responsive caregiving interventions. Lastly, the study was under-enrolled, as reflected by the width of the confidence intervals. 

Following community enthusiasm for the product, the multiple micronutrient-fortified beverage Chispuditos^®^ continues to be produced in Guatemala and is distributed in Guatemala, Nicaragua, and Honduras through a combination of grant-funded and direct-to-consumer distribution channels. 

## 5. Conclusions

Fortification is an important strategy to address micronutrient deficiencies. The finding that a multiple micronutrient-fortified beverage improved socioemotional development is consistent with other multiple micronutrient trials, warranting further investigation into the mechanisms by which micronutrients influence socioemotional development. Future studies targeting micronutrient deficiencies should measure socioemotional development. Although the theoretical underpinnings for combining multiple micronutrients and responsive caregiving interventions to support children’s development are strong, more careful integration and implementation strategies are needed.

## Figures and Tables

**Table 1:** Formulation of fortified beverages, Chispuditos^®^ and the placebo.

	Quantity per Serving ^a^	
	Chispuditos^®^	Placebo
Calories (Energy)	73 kcal	73 kcal
Protein	4 g	4 g
Fat	1 g	1 g
Dietary Fiber	2 g	2 g
Carbohydrates	12 g	12 g
Sugars	0 g	0 g
Micronutrients		
Iron	12 mg	-
Folic Acid	150 mcg	-
Zinc	9 mg	
Iodine	90 mcg	-
Vit A	250 mcg	-
Vit C	40 mg	-
Vit B12	0.9 mcg	-
Thiamine	0.5 mg	-
Niacin	6 mg	-
Riboflavin	0.5 mg	0.5 mg
Vit B6	0.5 mg	-
Copper	300 mcg	-
Vitamin D3	5 mcg	-
Vitamin E	5 mg	-
Calcium	200 mg	-
Phosphorus	150 mg	-
Magnesium	40 mg	-
Selenium	17 mcg	-
Manganese	0.17 mg	-
Biotin	8 mcg	-
Vitamin B5	1.8 mg	-

^a^ One serving = 18.75 g of dry product/day.

**Table 2:** Responsive feeding and caregiving key messages in English and Spanish for infants by age group (6–12 months, 12–18 months).

Age Group	Lesson Number	Message	Category ^a^	Picture ^b^
6–12 months	1	Smile and look at the child during feeding. Sonría y mire a su hijo(a) mientras lo(a) alimenta.	RF, RC	Mother and child looking, smiling, feeding
	2	Give your child opportunities to explore objects and people. Tummy time. Dele a su hijo(a) la oportunidad de explorar nuevos objetos, personas y alimentos. Es tiempo de estar boca abajo!	RC	Child on tummy playing
		Give your child family food of soft and thick consistency Alimente a su hijo(a)con comida de consistencia suave y espesa.	RF	Soft, thick food
	3	Eat and play with your child. Coma y juegue con su hijo(a).	RF, RC	Mother and child facing playing peek-a-boo, playing with household items
		Respond to your child’s cues of hunger and satiety. Reconozca las señales de hambre y saciedad de su niño.	RF	Mother feeding eager child
	4	Talk and sing to your child during daily activities. Háblele y cántele a su hijo(a) durante las actividades diarias.	RC	Mother singing and looking at child
		Have patience, love, and good humor when feeding your child. Cuando alimente a su niño tenga paciencia, amor y buen humor.	RF, RC	Mother feeding with a smile
	5	Imitate your child’s actions and gestures. Imite las acciones y gestos de su hijo(a).	RC	Mother imitating child
		Offer finger foods. Ofrezca alimentos que se puedan comer con la mano.	RF	Child picking up food with fingers
	6	Help your child learn signals, such as bye-bye. Ayude a su niño a reconocer señales, por ejemplo adiós.	RC	Child waving
		Feed your child an adequate amount and variety of food. Dele a su hijo(a) una alimentación apropiada, variada, especialmente de frutas y verduras.	RF	Mother feeding child; family meal
12–18 months	1	Offer finger foods. Déle a su niño alimentos que se puedan comer con la mano.	RF	Child picking up food with fingers
		Imitate your child’s actions. Imite las acciones de su hijo(a).	RC	Mother doing what child is doing
	2	Play with your child. Juegue con su hijo.	RC	Mother and child facing playing peek-a-boo, with a ball
		Respond to your child’s cues of hunger and satiety. Responda las señales de hambre y saciedad de su hijo(a).	RF	Child turning away from food, mother not forcing
	3	Establish a routine where you look at a book with your child. Establezca una rutina para observar un libro de cuentos con su hijo(a).	RC	Child in bed, mother with a book
		Eat in a calm, relaxed environment without distractions, such as TV or radio. Coma en un lugar tranquiloy sin distracciones.	RF	Family eating food together
	4	Talk and sing to your child throughout the day. Háblele y cántele a su hijo(a) a lo largo del día.	RC	Mother talking to child
		Take away uneaten food without comments. Retire los alimentos que el niño(a) no se comió sin decir nada.	RF	Mother picking up plate from child with food on it
		Talk to your child about daily household objects and activities. Hable con su hijo(a) acerca de objetos domésticos cotidianos y sobre actividades diarias.	RC	Child playing with household items
	5	Play matching games with your child. Juegue con su hijo(a) a buscar parejas (juegos de asociación).	RP	Mother and child looking at pictures in a book
		Make positive comments about the food (yummy). Haga comentarios positivos acerca de la comida.	RF	Mother smiling and eating with child
	6	Give adequate amounts of food at every meal. Dele a su hijo(a) cantidades adecuadas de alimentos en cada comida.	RF	Mother serving child
		Praise your child for good efforts even if he does not succeed. Felicite a su niño por los buenos esfuerzos a pesar de que no haya tenido éxito.	RC	Mother smiling at child
		Be sure that your child is hungry at meals and has not filled up on sweetened drinks or snacks. Asegúrese de que su niño tiene hambre en las comidas, y que no se haya llenado de bebidas dulces o golosinas no nutritivas.	RF	Child looking ready to eat

^a^ RC = responsive caregiving applied to play and learning, RF = responsive feeding. ^b^ Describes the picture that appears on the flip chart with each lesson.

**Table 3:** Baseline household, mother, and child characteristics by intervention group among infants in 72 community sectors (*n* = 386) ^a^.

Characteristic	Responsive Caregiving (*n* = 87)	Fortified Beverage (*n* = 98)	Responsive Caregiving + Fortified Beverage (*n* = 105)	Control (*n* = 96)	*p* ^b^
Household					
Family ethnicity, *n* (%) indigenous	14 (19%)	14 (17%)	10 (11%)	12 (15%)	0.59
Food insecurity, *n* (%)	52 (61%)	55 (59%)	55 (56%)	55 (60%)	0.90
Household assets, *n*	6.45 (2.66)	6.9 (2.93)	6.94 (3.14)	7.06 (3.02)	0.53
Household CHAOS score	14.12 (4.7)	15.3 (5.79)	13.49 (5.05)	14.33 (5.41)	0.12
Mother					
Age, y	27.62 (7.85)	28.76 (8.87)	26 (6.6)	27.84 (8.75)	0.10
Married/in a relationship, *n* (%)	75 (86%)	82 (84%)	93 (89%)	80 (83%)	0.69
<Primary schooling	17 (20%)	16 (17%)	20 (19%)	20 (21%)	1.0
Completed primary schooling	46 (53%)	50 (53%)	55 (52%)	49 (51%)	
≥Secondary schooling	24 (28%)	29 (31%)	30 (29%)	27 (28%)	
Child					
Age, months	12.87 (4.37)	12.78 (4.73)	13.19 (4.81)	13.02 (4.26)	0.93
Male sex, *n* (%)	43 (49%)	52 (53%)	49 (47%)	47 (49%)	0.84
LAZ	−1.6 (0.91)	−1.53 (1.05)	−1.37 (1.06)	−1.71 (1.01)	0.14
WAZ	−0.74 (0.93)	−0.79 (0.95)	−0.68 (0.92)	−0.96 (0.92)	0.20
WHZ	0.02 (0.93)	−0.02 (1.1)	0.01 (0.93)	−0.13 (0.95)	0.73
BMIZ	0.23 (0.94)	0.17 (1.15)	0.2 (0.96)	0.09 (0.94)	0.82
Child development					
Cognitive score	91.73 (11.21)	94.11 (11.55)	93.18 (12.19)	94.78 (12.22)	0.35
Language score	84.04 (15.31)	86.13 (14.73)	84.07 (15.6)	86.13 (14.21)	0.62
Motor score	85.86 (10.78)	87.46 (11.89)	86.17 (12.33)	88.34 (13.78)	0.49
Socioemotional competence	10.4 (5.67)	11.6 (5.64)	11.15 (5.97)	11.47 (6.05)	0.62
Socioemotional problems	17.1 (9.34)	19.7 (10.57)	16.87 (8.58)	17.86 (9.5)	0.21
Nutrition biomarkers					
Hemoglobin	10.98 (1.32)	10.86 (1.12)	10.61 (1.49)	10.76 (1.29)	0.58
Ferritin ^c^	10.4 (25.28)	12.48 (18.99)	8.05 (6.06)	11.29 (15.10)	0.05 *

^a^ Values are means (SDs) unless otherwise indicated. ^b^ *p*-value for baseline differences between groups from ANOVA. * *p* < 0.05. ^c^ Values are medians (IQRs). *p*-Value for baseline differences between groups from Kruskal–Wallis non-parametric ANOVA.

**Table 4:** Measures of child development and nutrition biomarkers by intervention group from the baseline to the endline ^a,b^.

	Wave	Fortified Beverage (*n* = 203)	No Fortified Beverage (*n* = 183)	Responsive Caregiving (*n* = 192)	No Responsive Caregiving (*n* = 194)	Fortified Beverage vs. No Fortified Beverage (95% CI) ^c^	Responsive Caregiving vs. No Responsive Caregiving (95% CI) ^c^
Child development							
Cognitive score ^d^	BL	93.63 (11.86)	93.32 (11.82)	92.51 (11.74)	94.44 (11.86)	−1.23 (−3.52, 1.06)	−0.59 (−2.88, 1.7)
	EL	88.76 (9.8)	89.97 (9.32)	88.79 (9.7)	89.85 (9.46)		
Language score ^d^	BL	85.08 (15.17)	85.13 (14.74)	84.05 (15.42)	86.13 (14.43)	0.13 (−2.71, 2.97)	0.06 (−2.78, 2.91)
	EL	82.98 (11.35)	82.78 (11.52)	82.66 (11.7)	83.09 (11.18)		
Motor score ^d^	BL	86.8 (12.1)	87.15 (12.47)	86.03 (11.62)	87.89 (12.82)	−0.14 (−2.65, 2.38)	−0.36 (−2.87, 2.16)
	EL	87.89 (11.3)	88.27 (12.13)	87.57 (11.59)	88.55 (11.8)		
Socioemotional competence ^e^	BL	11.35 (5.81)	10.98 (5.88)	10.83 (5.84)	11.53 (5.84)	2.34 (0.98, 3.7) **	0.17 (−1.19, 1.53)
	EL	11.97 (6.7)	9.61 (6.44)	10.96 (6.59)	10.7 (6.76)		
Socioemotional problems ^e^	BL	18.15 (9.61)	17.49 (9.4)	16.97 (8.91)	18.75 (10.05)	1.26 (−0.5, 3.02)	−0.83 (−2.59, 0.93)
	EL	11.05 (6.62)	9.64 (5.83)	9.86 (5.36)	10.84 (7)		
Nutrition biomarkers							
Hemoglobin, g/dL	BL	10.75 (1.3)	10.87 (1.3)	10.79 (1.42)	10.82 (1.19)	0.13 (−0.23, 0.49)	−0.15 (−0.51, 0.22)
	EL	11.07 (0.98)	10.98 (0.99)	10.95 (0.9)	11.09 (1.04)		
Ferritin, µg/L ^f^	BL	9.79 (14.85)	10.82 (19.31)	8.74 (12.65)	11.75 (17.1)	0.1 (−0.24, 0.45)	0.14 (−0.21, 0.49)
	EL	35.72 (87.3)	31.1 (76.73)	34.2 (76.13)	31.24 (90.5)		

^a^ Observed BL and EL values are means (SDs). BL, baseline; EL, endline; CI, confidence interval. ^b^ Multiplicative interactions between fortified beverage and responsive caregiving interventions were nonsignificant. Specific *p*-values for each dependent variable are as follows: cognitive score, *p* = 0.70; language score, *p* = 0.83; motor, *p* = 0.19; socioemotional competence, *p* = 0.44; socioemotional problems, *p* = 0.98. ^c^ Difference (95% CI) in change from the baseline to the endline in intervention vs. no intervention group. ** *p* < 0.01. ^d^ Cognitive, language, and motor scores were assessed using the Bayley Scales of Infant Development III. Cognitive scores are composite score equivalents. Language and motor scores are composite scores. Scores ranged from 40 to 160 with a mean of 100 (SD 15). ^e^ Socioemotional competence and problem scores were assessed using the Brief Infant-Toddler Social and Emotional Assessment. Competence scores ranged from 0 to 22. Problem scores ranged from 0 to 62. ^f^ BL and El values are median (IQR). Intervention vs. no intervention represents the geometric mean ratio of the relative change from the baseline to the endline comparing intervention groups with non-intervention groups.

## Data Availability

The data presented in this study is available upon request from the first or corresponding author. These data are not publicly available due to privacy issues.
